# Editorial: Flow cytometry - A powerful tool for diagnosis and therapy monitoring in hematology and immunology

**DOI:** 10.3389/fmed.2023.1282060

**Published:** 2023-09-13

**Authors:** Mihai Cenariu, Ravnit Grewal, Horia Bumbea, Daniela Sauma, Ciprian Tomuleasa

**Affiliations:** ^1^Department of Clinical Sciences, Faculty of Veterinary Medicine, University of Agricultural Sciences and Veterinary Medicine of Cluj-Napoca, Cluj Napoca, Romania; ^2^Department of Haematology, National Health Laboratory Services, Haematology, Gqeberha, South Africa; ^3^Department of Haematology, Faculty of Medicine, Carol Davila University of Medicine and Pharmacy, Bucharest, Romania; ^4^Department of Biology, University of Chile, Santiago, Chile; ^5^Department of Haematology, University of Medicine and Pharmacy Iuliu Hatieganu, Cluj-Napoca, Romania

**Keywords:** flow cytometry, diagnosis, therapy monitoring, hematology, immunology

*Flow Cytometry - A Powerful Tool for Diagnosis and Therapy Monitoring in Hematology and Immunology*, a Research Topic hosted by Frontiers in Medicine (Hematology), was launched in October 2021. The aim was to offer a platform to different research groups, that use flow cytometry for diagnosis and therapy monitoring in hematology and immunology, to present their valuable work and results. A total of five original research articles were published, together with two case reports and one review article. The topics were diverse, but connected by flow cytometry, which was the core method employed by all research teams.

Hiza et al. used flow cytometry to measure the expression of CD38 or CD27 on CD4+ T cells producing interferon gamma and/or tumor necrosis factor alpha in patients with tuberculosis. This disease is still one of the main causes of morbidity and mortality due to an infectious agent, despite being treatable using a combination of drugs (1). Moreover, monitoring the course of the disease in each patient is challenging and development of drug-resistant tuberculosis remains a hazard to the public (2). Therefore, this study aimed at investigated whether CD4+ T cells activation markers such as CD38 and CD27 could be correlated to disease severity and assess the response to treatment. The results showed a higher ability of CD38 to distinguish active tuberculosis patients from healed individuals compared to CD27. Additionally, CD38 was not quantitatively induced by the presence of live mycobacteria recovered from the patient's sputa at the time of diagnosis. Thus, this study showed that a CD38-based assay could be a method to monitor treatment response.

Baldasso et al. developed and validated a flow cytometry antibody test against *Lawsonia intracellularis*-induced IgG in swine, with a sensitivity of 98.8% and specificity of 100%. Porcine proliferative enteropathy (PPE) is an intestinal illness with a significant economic effect on pig industry and is produced by *Lawsonia intracellularis*, an obligate intracellular bacterium (3). In order to assess the infection dynamics, type and extent of passive immunity, as well as strength and duration of vaccine-induced antibody response, serological tests such as ELISA are nowadays available but with low sensitivity (4). The assay developed herein uses whole, live-attenuated *L. intracellularis* bacteria derived from a commercial vaccine; thus, it is highly recommended for seroepidemiological studies, evaluation of infection dynamics and characterization of the humoral response following vaccination.

Jiang et al. performed flow cytometric analyses of blood samples in a cohort of nine patients that underwent chimeric antigen receptor T (CAR T) cell therapy coupled with granulocyte-macrophage colony-stimulating factor, in an attempt to improve neutropenia, uphold recovery of cellular immunity, and boost CAR T-cell expansion. CAR T cell therapy is currently being used as a treatment option in several hematological cancers. Nevertheless, its effectiveness is sometimes limited due to insufficient persistence within the host and multiple side effects (5). Following a median intervention time of 15 days, CAR T-cell expansion was observed in the peripheral blood of seven patients. Additionally, all patients had increased white blood cell counts as well as neutrophiles, lymphocytes and CD3-CD16+CD56+ natural killer cells occurred in all patients. There was no fatal infection, nor a cytokine release syndrome in this study.

Cai et al. investigated by flow cytometry the T-lymphocyte subsets (CD4+, CD8+) and T-lymphocyte activation (CD69+, CD25+, HLA-DR+) in the peripheral blood of children with Hodgkin's lymphoma (HL), to identify the potential prognostic factors for event-free survival. HL is one of the most frequent tumoral diseases diagnosed worldwide. Recent studies show that HLA-DR+/CD38 T cells may be related to relapse and refractoriness in pediatric HL (6). Moreover, CD25+ (7) and CD69+ (8) cells were identified in the tumor microenvironment. They concluded that the peripheral immune status may be related to disease severity in HL. Thus, CD3+CD4+HLA-DR+ T cells and CD3+CD8+HLA-DR+ T cells may be a novel indicator for risk stratification of HL and an independent risk factor for inferior outcome in childhood HL.

Cianga et al. used flow cytometry to investigate peripheral blood NK cells of acute myeloid leukemia (AML) patients in terms of numbers, distribution across maturation stages or inhibitory receptors expression. They correlated this information with the genetic background offered by killer immunoglobulin-like receptors (KIRs) and the HLA-C genotypes. AML is a hematological malignancy that is heterogeneous, clinically, morphologically as well as genetically. It results from the clonal expansion of blasts of any of the myeloid lineages in the peripheral blood, bone marrow as well as other tissue (9). Previous research proved that anti-leukemic natural killer (NK) cells are reduced in numbers and they present a reduced receptor expression while their associated ligand is also downregulated (10). This study provided key information regarding receptors targeted as checkpoint inhibitors in immunotherapy. It was shown that AML patients with complex karyotypes or displaying a FLT3 gene mutation, had very low NK cells percentages or high expression of inhibitory receptors.

Popa et al. presented a case report concerning a pediatric patient with T-acute lymphoblastic leukemia in which flow cytometric immunophenotyping at diagnosis and during treatment was performed. Despite the various therapeutic measures that were used which included a stem cell allotransplantation, a chemoresistant clone persisted.

Tadros et al. presented a case report where the early diagnosis of a hairy cell leukemia was possible by flow cytometric analysis of a small leukocyte population that exhibited a higher side scatter and brighter CD19/CD20. A total of 1,000,000 events were analyzed, which allowed a better study of the neoplastic subpopulation. Thus, flow cytometric analysis enabled the therapeutic decision and disease progression toward remission.

Last, but not least, Munteanu et al. reviewed the flow cytometric evaluation of the immunological effects of several vitamins, such as: the role of vitamins E in the prevention and treatment of different types of cancer, the properties of K vitamins in the development and maintenance of PC12 cells in Parkinson's disease, the effect of vitamin B5 on the loss of bone mass in low estrogen conditions, the anticancer role of vitamins B6, the role of Vitamin B9 in the regulation of Treg cells.

## Author contributions

MC: Conceptualization, Writing—original draft. RG: Conceptualization, Formal analysis, Writing—review and editing. HB: Conceptualization, Formal analysis, Writing—review and editing. DS: Conceptualization, Formal analysis, Writing—review and editing. CT: Conceptualization, Formal analysis, Writing—review and editing.
